# Economic Decision-Making in Parrots

**DOI:** 10.1038/s41598-018-30933-5

**Published:** 2018-08-22

**Authors:** Anastasia Krasheninnikova, Friederike Höner, Laurie O’Neill, Elisabetta Penna, Auguste M. P. von Bayern

**Affiliations:** 1 Max-Planck-Institute for Ornithology, Eberhard-Gwinner-Str., 82319 Seewiesen, Germany; 2 Max-Planck Comparative Cognition Research Station, Loro Parque Fundación, 38400 Puerto de la Cruz, Tenerife Spain; 3 Biozentrum Grindel and Zoological Museum, Martin-Luther-King-Platz 3, 20146 Hamburg, Germany; 4 Department of Life Science and Systems Biology, University of Turin, Via Accademia Albertina 13, 10123 Turin, Italy; 5 Department of Biology, Ludwig-Maximilians-University of Munich, Großhaderner Str. 2, 82152 Planegg-Martinsried, Germany

## Abstract

Economic decision-making involves weighing up differently beneficial alternatives to maximise payoff. This sometimes requires the ability to forego one’s desire for immediate satisfaction. This ability is considered cognitively challenging because it not only requires inhibiting impulses, but also evaluating expected outcomes in order to decide whether waiting is worthwhile. We tested four parrot species in a token exchange task. The subjects were first trained to exchange three types of tokens for a food item of low, medium, and high value and successfully learned to exchange these in an order according to their value. Subsequently, they were confronted with a choice between a food item and a token that could be exchanged for higher-quality food. In additional control conditions however, choosing a token led to an equal or lower payoff. Individuals of all species were capable of deciding economically, yet only large macaws outperformed the other species in one of the crucial controls. For some individuals, particularly African grey parrots, the token apparently had an intrinsic value, which prevented them from choosing economically in some control conditions and which should be considered as potentially confounding by future token exchange studies.

## Introduction

Economic and evolutionary theories of human and animal decision making have much in common as both focus on profit maximisation^1,2^. In fact, when faced with options available at different times, so-called intertemporal choices, individuals must sometimes forgo their desire for immediate satisfaction to obtain a higher payoff in the future. Such self-control is considered cognitively challenging because the subjects not only have to suppress impulsive reactions but also must assess and compare the values of different options so as to decide whether or not an immediate option is worth sacrificing.

In classical delay of gratification tasks, subjects are given a choice between an immediate reward of low value and a delayed one of higher value^3^. Typically, once the choice is made the decision cannot be reverted and the subject must wait for the whole duration of the delay before obtaining their larger reward. Other methods measure how long subjects can wait by allowing them to change their mind and interrupt the delay. This is the case in “delay maintenance” tasks such as accumulation tasks where subjects can stop a steady accumulation of food rewards by reaching for them, and in “delayed exchange” tasks where individuals endowed with an initial low value item can give up (consume the item) at any time during the delay prior to being asked to exchange it for a food of higher quality or quantity^4,5^. The latter has also been commonly used to investigate economic decision-making because it mimics an economic transaction^6,7^.

A wide variety of exchange and choice tasks has shown that nonhuman primates can maximize their future profits by forgoing immediate benefits. For example, chimpanzees (*Pan troglodytes*) exchange small quantities of food for larger quantities^8^ and brown capuchin monkeys (*Sapajus spp*.) trade low-value foods for high-value foods^9^ and forego consuming an immediately available piece of food if the food serves as a token to obtain more valuable food at a later point in time^10^. The capacity to optimise one’s future yield has also been reported from non-primate species such as dogs^11^, sea lions (*Zalophus californianus*)^12^, cleaner wrasses (*Labroides dimidiatus*)^13^ and more recently also from large-brained birds such as corvids^14,15^ and parrots^16–18^. However, the reported performance in the delayed exchange paradigm across and within taxa are rather mixed. The tolerance of a delay that allows a future profit maximisation ranges from seconds to minutes depending on, for example, whether the subjects were asked to maximize quantity or quality of the yield^15,16,19^. From a comparative perspective, some methodological issues have been pointed out, particularly concerning the comparison between primates and other species. For example, when testing nonhuman animals that do not have functional hands such as birds or dogs, the subjects are typically required to keep the tradable food item in their beak or mouth. However, holding food in contact to the taste organs probably makes it more difficult to inhibit the impulse to eat the initial item^11,19^. Another issue that has resulted in ambiguity is that subjects in some studies could eat some of the reward before exchanging it, thus both easing their waiting time and increasing their pay-off. If the food item had to be returned intact, some animals could no longer delay the exchange or exchange less preferred items for more preferred ones^9,20^.

An elegant solution to resolve these issues has been implementing tokens as symbolic representations of food that could be traded for rewards as a kind of currency^21–25^. In this manner, subjects do not have to wait with or forego food already in their possession. Symbolic representations of food may aid a subject because it does not have to override appetitive features of an initial reward that serves as a token, thus achieving psychological distancing. Substituting real food items with symbolic representations indeed turned out to improve the subjects’ performance in primates^26^. However, these primate studies offered a binary choice between non-edible objects and it remains debatable whether it is challenging in terms of self-control^27^. An alternative approach is offering a token exchange possibility that involves a choice between immediately available food and a token that can be exchanged for a higher-value food item in an additional exchange. The advantage of this approach is that it helps to avoid both psychological distancing from the actual object of desire (food) and possession of food during the delay. To date, only a few studies have directly pitted tokens representing different types of food against physically present food^27,28^. For example, Beran and Evans^27^ gave chimpanzees a binary choice between a food item and a token that could be exchanged for a higher-quality food item. The chimpanzees tended to select an additional exchange rather than the immediately available food. Similar results were reported by Judge and Essler’s^28^ study on capuchin monkeys.

In birds, no study has used the token exchange methodology to test economic decision-making. Nevertheless, there is compelling evidence from a study comparable to the token exchange paradigm, but in a tool-use context, that Goffin’s cockatoos (*Cacatua goffini*), a distinct lineage (superfamily *Cacatuoidea*) within the order psittaciformes, can make profitable decisions^29^. The tool-use trained birds had to choose between immediately available food or a tool required for accessing a more valuable reward. Making their choice they had to consider both the quality of the immediately available versus the delayed food and the functionality of the provided tools. These findings suggest that psittaciformes are promising candidates for studies of economic decision-making. Given that tool use in parrots is exceptional, it is essential to use a more general, simpler paradigm such as the token exchange task that can be applied in a broader comparative context.

In the present study, we investigated economic decision-making in four parrot species from the family Psittacidae, thus belonging to another psittaciform superfamily (*Psittacoidea*) than the distantly related cockatoos. They were presented with a binary choice between an immediately available food reward or a token that could be exchanged for another (or the same) food reward in the future. We used three differently preferred food rewards (low-, medium- and high-quality; see methods) associated with three different tokens. We investigated whether the parrots could learn to associate different tokens with different types of food and would select a token for a food reward over an immediately available food of lesser value. If the parrots were capable of deciding economically, i.e., maximising their pay-offs, we would predict them to choose the most profitable option (food or token), while avoiding unnecessary effort. Some predictions could be made about how the different species in the current test would perform. Some initial data on the feeding ecology of the species are available that would suggest that the great green macaws (*Ara ambiguus*) and the blue throated macaws (*Ara glaucogularis*) were feeding specialists, relying heavily on mountain almond trees and motaçu palm fruits respectively^30,31^ which can be considered as ephemeral food sources. Furthermore, the African grey parrots (*Psittacus erithacus*) are observed to be more generalist feeders with a more granivorous diet consisting of various types of seeds and nuts^32^ and finally the blue-headed macaws (*Primolius couloni*) are relatively unknown in their feeding styles but are thought to move in a nomadic style that suggests no particular reliance on an individual food resource (except clay licks)^33^ and therefore also are likely to be feeding generalists. With this in mind, it is possible to hypothesise that the feeding specialists (the great green and the blue-throated macaws) might encounter a greater need for optimal decision-making as to where and when to go more often than feeding generalists (the African grey parrots and the blue-headed macaws), who may opportunistically take any food they encounter. Therefore, we predicted that the feeding specialists would be more prone to making optimal decisions.

We tested the parrots in the conditions implemented by the previous primate studies but also applied additional test and control conditions. In three conditions the subject could increase its payoff by choosing the token over the immediately available food, whereas in one condition, choosing the token led to a lower payoff than choosing the food. Finally, two conditions presented a situation where there was no difference between the immediate and the future gain, so that the most economic decision was to consume the food immediately. The primate studies mentioned above^27,28^ have incorporated some of these control conditions but missed others, thus providing an incomplete picture of the subjects’ motivations when selecting tokens over food rewards and their abstract representation of the options’ value.

## Results

The main findings are summarised in Table 1 showing the number of individuals per species who made optimal choices (token or food) across conditions. The conditions displayed in Table 1 are described below.

**Table 1:** Number of individuals performing above chance level (15 or more out of 20 trials) across the six test conditions.

Condition	Great green macaws (N = 9)	Blue-throated macaws (N = 8)	Blue-headed macaws (N = 8)	African grey parrots (N = 6)
1 LT (LF vs HT)	9	8	8	6
2 MT (MF vs HT)	9	8	8	6
3 LT (LF vs MT)	7	4	3	5
4 MT (MF vs LT)	9	7	8	6
5 MT (MF vs MT)	7	6	8	3
6 HT (HF vs HT)	9	8	2	2

N = number of subjects tested per species.

### *Condition 1* Low Token→ Low Food versus High Token = LT (LF vs HT)

When faced with a choice between the low-value food (dry corn) and the token that could be exchanged for the high-value food (walnut piece), all subjects chose the token, thus the more advantageous option, in all 20 trials, except one blue-headed macaw, who took the dry corn in one trial (binomial test, all p < 0.05).

### *Condition 2* Medium Token→ Medium Food versus High Token = MT (MF vs HT)

When confronted with a choice between the medium-value food (sunflower seed) and the token that could be exchanged for the high-value food (walnut piece), all subjects still chose the token significantly above chance level (binomial test, all p < 0.05), although the difference in value between the rewards was less pronounced than in *condition 1*.

### *Condition 3* Low Token→ Low Food versus Medium Token = LT (LF vs MT)

In the third test condition, we replaced the high value token with the medium value one, to examine whether the birds were also willing to forego their reward for a token, if it was not the high-value token. When choosing between the low-value food (dry corn) and the token for the medium-value food (sunflower seed), seven out of nine great green macaws, four out of eight blue-throated macaws, three out of eight blue-headed macaws and five out of six grey parrots, selected the token significantly above chance level (binomial test, all p < 0.05). One blue-throated macaw and two blue-headed macaws chose the token significantly less than chance level (binomial test, all p < 0.05). All other individuals performed at the chance level (binomial test, all n.s.).

### *Condition 4* Medium Token→ Medium Food versus Low Token = MT (MF vs LT)

In the first condition controlling against a general preference for tokens, the birds could select between a piece of medium-value food (sunflower seed) and the token against which a piece of the low-value food (dry corn) could be traded. All birds but one blue-throated macaw chose optimally by selecting the sunflower seed significantly above chance level (binomial test, all p < 0.05).

### *Condition 5* Medium Token→ Medium Food versus Medium Token = MT (MF vs MT)

In the second control condition for a token preference, in which the birds could choose between the medium-value food (sunflower seed) and the token for the medium-value food, seven great green macaws and six blue-throated macaws, respectively, all eight blue-headed macaws, and three grey parrots avoided the delayed pay-off by selecting the immediately available medium-value food rather than the medium-value token (binomial test, all p < 0.05).

### *Condition 6* High Token → High Food versus High Token = HT (HF vs HT)

**Figure 1:**
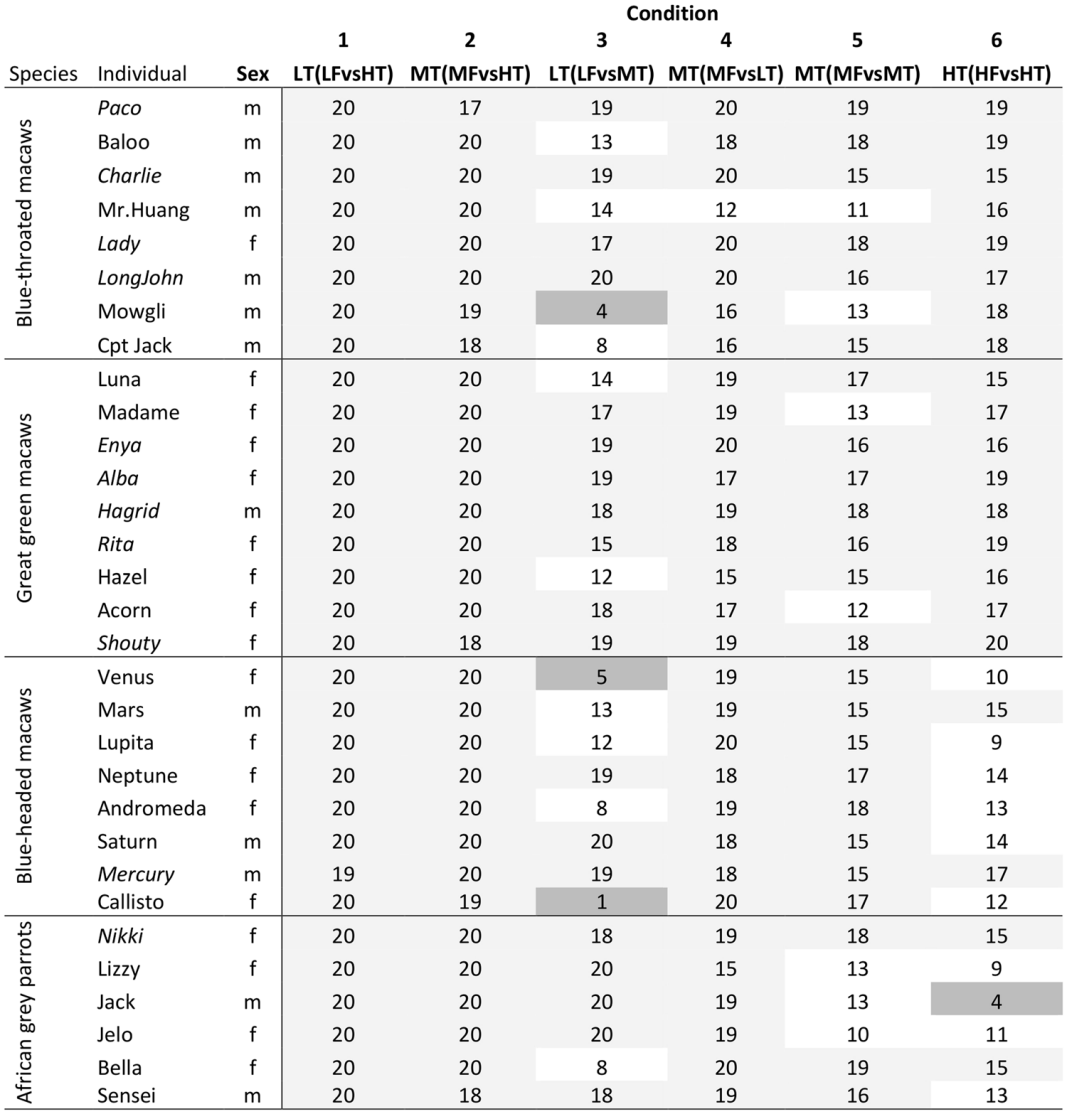
The number of choices (out of 20) in which an individual chose the item (token or food) that constituted the optimal choice. The performances significantly above chance level (according to a Binomial distribution 15 or more out of 20 trials) are highlighted in light grey shading, and those significantly below the chance level (5 or less out of 20 trials) in dark grey. Individuals that performed above chance level in all six test conditions are marked in italics.

In the third control condition, in which the birds were confronted with a choice between the high-value food (walnut) and the token for the same high-value food, all great green macaws and blue-throated macaws, but only two blue-headed macaws and two grey parrots avoided the extra effort by selecting the high-value food (walnut) directly (binomial test, all p < 0.05). One grey parrot on the contrary had a strong preference for the token choosing the food significantly less than chance level (binomial test, p < 0.05, see Fig. 1).

**Figure 2:**
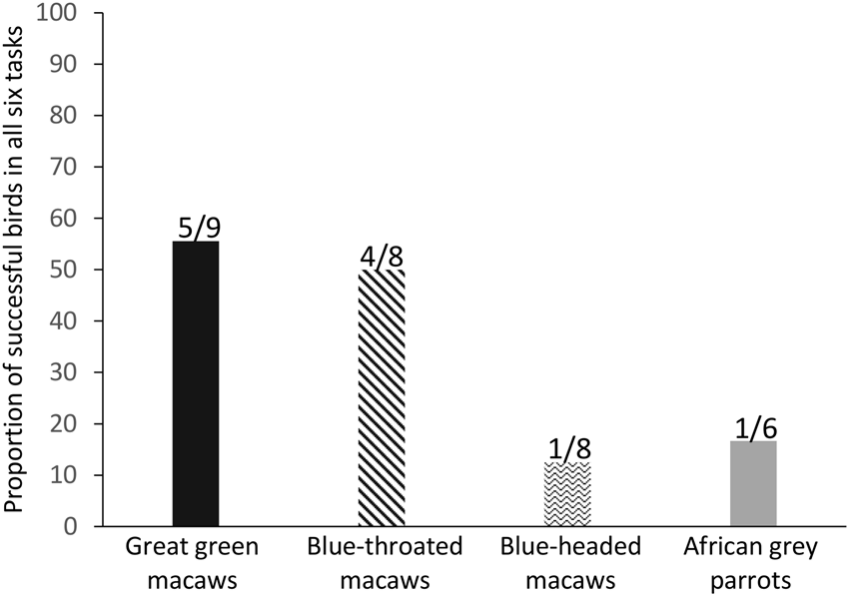
The proportion of individuals per species that selected the item (token or food) that yielded maximum payoff in all six test conditions.

At an individual level, five out of nine great green macaws, four out of eight blue-throated macaws, one out of eight blue-headed macaws, and one out of six grey parrots selected the most profitable option across all six conditions (see Fig. 2).

**Figure 3:**
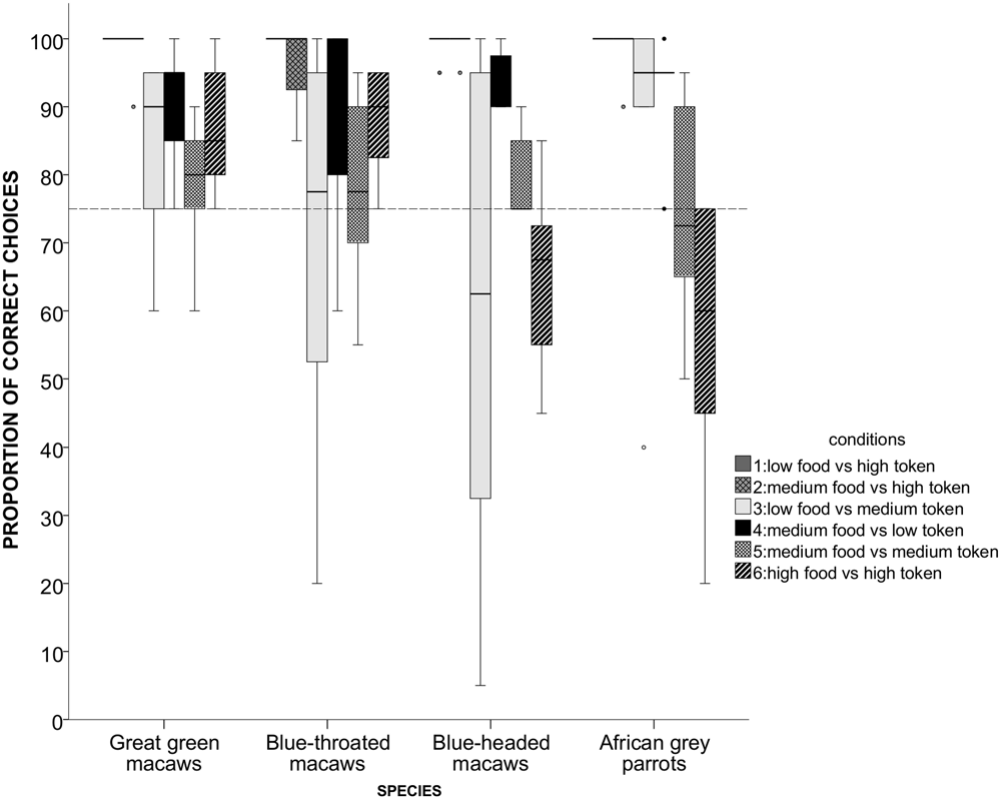
The mean proportions of “economic” choices per condition across species. The dashed line indicates the chance level on the individual level; circles represent outliers. The *condition 1*, *2*, and *3* represented a choice between a food item and a token that could be exchanged for higher-quality food, where condition 3 was a control for an intrinsic value of the high-value token. In the other control conditions, the choice of a token led to lower (*condition 4*) or an equal (*condition 5* and *6)* payoff. Boxes show the interquartile range from the 25th to the 75th percentile. The line across the boxes represents the median. The whiskers indicate the maximum and minimum values excluding outliers (circles).

At a group level, the great green macaws were the only species in which most individuals performed significantly above chance level in all test conditions (Fig. 3). Compared to them, the blue-throated macaws showed slightly greater individual variation in their performance across the test conditions. Most of the blue-headed macaws failed to select the most advantageous option in test *condition 3* LT (LF vs MT) and control condition *6* HT (HF vs HT), and the majority of the grey parrots failed to do so in both conditions in which they faced a choice between a food and a token of equal value, i.e. in *condition 5* MT (MF vs MT) and *condition 6* HT (HF vs HT).

Looking at species differences in the different conditions, we found that species differed significantly only in the control *condition 6* HT (HF vs HT; Kruskal-Wallis test, Chi^2^ = 19.332, p < 0.001). A post-hoc test revealed that the great green macaws and the blue-throated macaws selected the immediately available piece of walnut significantly more often than the blue-headed macaws and the grey parrots (Dunn-Bonferroni-Tests, great green macaws vs blue-headed macaws, p = 0.018 and great green macaws vs grey parrots, p = 0.009; blue-throated macaws vs blue-headed macaws, p = 0.007 and blue-throated macaws vs grey parrots, p = 0.014).

## Discussion

The parrots of all four species (except for two grey parrot individuals that therefore did not take part in the subsequent tests) selectively chose tokens associated with more preferred foods first, handing the provided tokens back in a predictable order according to preference. These findings parallel earlier findings in primates^21,24^ and suggest that the parrots indeed attributed value to the tokens. When provided with a choice between a food or a token that could be exchanged for more preferred food, all four species inhibited their impulsive reactions and selected the token significantly more often than chance, thus maximising their payoff. They did so regardless of the value of the immediate gain, i.e., low (*condition 1*) or medium (*condition 2*). In fact, their performance in *condition 1* was comparable to that of chimpanzees (with three out of three chimpanzees selecting the token over the food to increase their yield)^27^ or even slightly better than that of capuchin monkeys (with two out of four monkeys choosing economically)^28^ or Goffin cockatoos (five out of 13 birds selecting the tool that led to a higher payoff)^29^. Note that all four capuchin monkeys in the Judge and Essler study^28^ failed to maximise their payoff when faced with the choice between medium-value food and high-value token (*condition 2* in the present study), although one individual succeeded in a later re-test. Taken together, our results strengthen previous findings of high level of inhibitory control in parrots in delayed gratification tasks^16,17^, despite their apparently poor motor self-regulation performance when tested in a detour reaching task^34,35^.

More critically, when facing the choice between food and a token through which they could obtain food of lesser value (*condition 4*), all birds of all four species (except for one blue-throated macaw individual) selected the immediate food reward over the token, even if it was not the highly preferred food. These findings are in line with the previous results found in primates and suggest that the parrots did not merely learn a “select a token over food” rule. In contrast to the study on chimpanzees, which had to choose between the low-value token and high-value food^27^, the parrots and previously tested capuchins monkey^28^ faced an arguably more difficult choice in this condition, because the discrepancy in value between the two options was less pronounced (parrots and capuchins: MF vs LT, compared to chimpanzees: HF vs LT). When a medium-value token was offered as the alternative to the low-value dry corn (*condition 3*) however, some individuals of the blue-throated and the blue-headed macaws had more difficulty to decide economically and picked randomly. The higher number of mistakes in this condition suggests that either the high token had an intrinsic value for them, or the difference between the immediate and the future gain was not sufficiently pronounced to overcome their desire for immediate satisfaction.

The remaining two controls presented situations where the immediate and future yield was of the same quality to test whether the subjects would avoid the unnecessary effort and extra waiting time by selecting the immediate food reward. The majority of the grey parrots performed equally poor in both conditions and unnecessarily chose the token suggesting that they merely learnt a “select a token over food” rule. However, the outstanding performance of the grey parrots in the *condition 3* MT (MF vs LT), where choosing the token would result in obtaining a food of lesser value contradicts this assumption. Similarly contradictorily, the blue-headed macaws performed well when faced with a choice between token and food of the medium value (*condition 5*), but failed to optimise their choices when faced with the most preferred food (a piece of walnut) and its associated token (*condition* 6). This may imply that the high-value token had acquired an intrinsic value for them, and the same might be true for most of the grey parrots. From a comparative perspective, the large macaws performed comparably to chimpanzees, which in the Beran and Evans (2012) study always selected food over lexigram tokens representing the same food^27^. Overall the parrots’ performance in these two conditions compared to that of Goffin cockatoos tested in a comparable situation. Five of 13 Goffin cockatoos selected their most preferred food over a tool that could be used to obtain the same food^29^ (an analogue to *condition 6* in the present study).

In summary, on a group level, the great green macaws were the only species in which most of the individuals performed significantly above chance throughout all six test conditions, suggesting that they successfully weighed up between the two options to make the most advantageous choice. These findings are in line with previous studies showing that cockatoos, a distantly related psittaciforme, also decide economically. Considering that the blue-throated macaws performed well overall but showed a weaker performance only when the token offered was of fairly low value compared to the immediate reward (*condition 3*), we assume that motivational issues might have confounded the results. For them, the difference between the immediately available corn and the potential sunflower seed, which they could have traded the medium-value token for, might not have been sufficiently pronounced to justify the extra effort/additional waiting time of another exchange. In contrast to the larger macaws, the blue-headed macaws appeared to generally prefer the high-value token over the immediate food, even if it was of the same value. We therefore cannot exclude that they might have followed the rule “always take the high token, otherwise take the food”. Alternatively, however, their economic decision-making ability might have been confounded because they appeared affected by the intrinsic value of the high-value token. The majority of the African grey parrots failed to optimise their choice only when the token was of the same value than the immediately available food, i.e. in *conditions 5* and *6*. Here they often chose the token despite the unnecessary effort. One possible reason for their uneconomic behaviour may be that they found interacting with the higher value tokens as self-rewarding and thus might not have perceived trading them as a notable effort. This is not unlikely, given that some parrot species have strong tendencies to engage in object play^36^. It would be interesting if future studies tested whether increasing the costliness of the token exchange (e.g., prolonging the waiting time before exchange or adding effort) would change their behaviour.

In our study, the two larger macaw species stood out in the number of individuals that chose optimally in *all* six test conditions. Specifically, they outperformed the others in control *condition 6*, thus suggesting that they were more likely to be deciding economically compared to the smaller blue-headed macaws and the grey parrots, who may have used a simpler rule to always choose the high token. A species’ feeding ecology has often been suggested as one of the selective forces affecting inhibitory control and evolution of economic decision-making^35,37,38^. The great green macaws and the blue-throated macaws are supposedly feeding specialists with pronounced dependence on nuts and fruits, thus ephemeral food sources^31,39^, while the blue-headed macaws’ and African grey parrots’ diets have a broader spectrum among our tested species^32^. However, ecological explanations for this difference are highly speculative and the exact cognitive demands of the species’ feeding ecology are a matter of ongoing dispute and can be viewed in diverse ways^35,38,40^. For example, one might consider the ephemeral nature of the larger macaws’ main food sources less demanding in terms of the need for pay-off maximisation since the birds should always exploit any limited food source they encounter^38,40^. However, our data stand in contrast with this assumption. On the other hand, one may argue that economic decision making might play a greater role in feeding specialists than more generalist and thus opportunistic species, as they may have to weigh up when to leave one food source in order to reach the next in time, in which case our data will be in line with this expectation. Whatever argumentation one might be willing to follow, one should keep in mind that the foraging behaviour of most wild parrots is very difficult to assess and any correlations between economic decision-making and feeding ecology should be considered with precaution until more data become available.

Another factor that ought to be considered when construing the species’ differences in the performance may be a minor methodological difference in the order of test conditions. The African grey parrots, one blue-throated macaw (Mowgli) and one great green macaw (Shouty) were administered trials of the six different conditions within the same session, whereas all the other birds received the trials of *conditions 1*, *2*, *4* and *5* and the two control conditions (*3* and *6*) in separate sessions. However, no pattern was found suggesting that the species’ performance was affected by having conditions administered in two different sessions. For instance, the blue-headed macaws, which can be considered as the worst performing species, mostly failed in the two control conditions (*3* and *6*) that were administered separately from the other four conditions, while the great green macaws performed equally well in the sessions with the four conditions (*1*, *2*, *4* and *5*) and in the sessions with the two additional controls (*3* and *6*). The blue-throated macaws failed in the control *condition 3*, but not in the *condition 6*, even though both were administered separately from the other four conditions. Also, among the birds that were administered trials of the six different conditions within the same session, no pattern suggesting that the testing order of conditions had played a role was observed. While the grey parrots performed particularly poorly in *conditions 5* and *6*, the blue-throated macaw (Mowgli) failed in *conditions 3* and *5* and the great green macaw (Shouty) performed exceptionally well in all six conditions.

In summary, the parrots in the present study inhibited their impulsive reactions if it led to an increased payoff. Particularly, the performance of the two larger macaws was equally good or even better than the performance of primates and cockatoos in comparable conditions, and most importantly, in the critical controls, suggesting that they have the capacity to decide economically and optimise their behaviour. The blue-headed macaws and African grey parrots inhibited their impulsive reactions by choosing tokens over food but failed to choose economically in some of the control conditions. We suggest that their capacity to choose economically might have been affected by certain confounding factors as outlined above that ought to be carefully considered by future token exchange studies.

## Material and Methods

### Subjects

We tested 36 individuals of four different parrot species: Nine great green macaws (eight females, one male, mean age 3.33, SD = 2.69, see Fig. 1), eight blue-throated macaws (one female, seven males, mean age 3.13, SD = 2.03), eight blue-headed macaws (five females, three males, all two years old), and eight African grey parrots (six females, two males, all two years old). All parrots had been hand-raised under comparable conditions and subsequently socialised in groups in the Loro Parque Fundación, Tenerife, Spain.

### Housing conditions

All parrots were housed in aviaries at the Max-Planck Comparative Cognition Research Station located within the Loro Parque in Puerto de la Cruz, Tenerife. The blue-throated macaws and the great green macaws were housed in eight aviaries, divided by species and age into five groups of two to eight individuals. Six of these aviaries were 1.8 m × 3.4 m × 3 m (width × length × height), and the remaining aviaries were 2 m × 3.4 m × 3 m and 1.5 m × 3.4 m × 3 m, respectively. These aviaries were interconnected by 1 m × 1 m windows, which could be closed when desired. The blue-headed macaws were housed together in a separate indoor area (28.61 m^2^) with access to a smaller outdoor area and the African grey parrots were housed together in another separate outdoor aviary (21.41 m^2^). All aviaries had at least one side open to the outside, so they followed a natural light schedule and were also kept to ambient Tenerife outdoor temperature, on average between 17 and 25 degrees Celsius, but they were additionally lit with Arcadia Zoo Bars (Arcadia 54 W Freshwater Pro and Arcadia 54 W D3 Reptile lamp) to ensure sufficient exposure to UV light. They were also all within the same site as the testing chambers (described below).

### Experimental setup and procedures

Training and testing took place in an indoor area equipped with lamps covering the birds’ full range of visible light (Arcadia 39 W Freshwater Pro and Arcadia 39 W D3 Reptile lamp). The testing area consisted of two rooms, each 2.5 m × 1.5 m × 1.5 m (height × width × length), separated by a window (1 m × 1 m) with an opening that could be covered with different plastic panels (depending on the experiment). Each testing room was equipped with a table and a wooden multi-step perch.

The subjects were individually tested in one of the testing chambers with the experimenter in an adjoining room. A sound-buffered one-way glass system permitted zoo visitors to see inside the rooms, but did not allow the birds to see out. All training and testing sessions took place either in the morning or in the afternoon, a minimum of 4 h after the last feeding (or overnight for morning sessions). The parrots were fed twice a day with a mix of fruits and vegetables shared between all the birds, and in the evening, after the test sessions, they received an individual amount of seeds calculated considering the amount of rewards (walnuts and seeds) they had received during the tests and their individual weight. Water was available *ad libitum*.

All the subjects had been tested before in different kinds of social and physical cognition tests, so they were well habituated to the testing rooms, as well as to be moved to them, and they were accustomed to interact with human experimenters. All birds were also familiar with the token exchange procedure from a previous study (Krasheninnikova *et al*. unpublished data).

### Training

Before the experiments began we performed a food preference test following Judge and Essler’s^28^ procedure (see Supplemental Information, SI hereafter) to establish three food items of different values: a piece of walnut as a highly preferred food, one sunflower seed as a medium-value reward, and one kernel of dry corn as the low-value reward. Subsequently, the birds learned to associate these three food items with particular tokens; a piece of plastic pipe for the high-value food, a metal bracket for the medium-value food and a metal loop for the low-value food (see Fig. S1). The birds were presented with three tokens of each type, nine in total, placed in the testing room in a random arrangement. The assumption was that if the birds had learnt to associate tokens with particular types of food, they would first hand out the three tokens associated with the most preferred food followed by the three tokens associated with the medium-value one, and finally exchange the three tokens associated with the low-value corn. To meet criterion for testing, they had to exchange tokens in this order throughout three consecutive sessions. The majority of the birds learned the association within 15 sessions, and two African grey parrots failed the association training after 35 sessions and were excluded from the experiment (see Table S2).

### Testing

After reaching the training criterion the subjects proceeded to the six test conditions (see Table 2). Two trials of each condition were presented in a pseudo-randomised and counter-balanced manner within the same session (ten sessions in total) to control for order or/and experience effects. For the grey parrots that were tested last, as well as one blue-headed macaw (Mowgli) and one great green macaw (Shouty) tested later than their conspecifics because of health issues, this included *condition 3* and *condition 6*, hence 12 trials per session, whereas for the other three species these two conditions were added after they had been tested in *conditions 1*, *2*, *4*, and 5. Therefore, the blue-throated macaws, the great green macaws and the blue-headed macaws first received 10 sessions of eight test trials per session (i.e. two trials of *conditions 1*, *2*, *4*, *and 5*), followed by 10 sessions of four test trials per session (i.e. two trials of *conditions 3* and *6*).

**Table 2:** Overview of the six test and control conditions.

Condition	Initial token	Choice between	Economic decision	
1 LT (LF vs HT)	Low	Low Food	High Token	Token
2 MT (MF vs HT)	Medium	Medium Food	High Token	Token
3 LT (LF vs MT)	Low	Low Food	Medium Token	Token
4 MT (MF vs LT)	Medium	Medium Food	Low Token	Food
5 MT (MF vs MT)	Medium	Medium Food	Medium Token	Food
6 HT (HF vs HT)	High	High Food	High Token	Food

Following the methodology from Judge and Essler^28^, the general testing procedure consisted in placing an initial token in the birds’ testing compartment and requesting it back with an open palm gesture. Once the bird returned the initial token into the experimenter’s open palm, the experimenter removed the hand with the initial token and immediately offered a choice between two hands with palms facing up, one holding the food reward associated with the initial token the other holding another token, associated with the higher, the same or the lower food reward, depending on the condition. The subjects could not see what they could exchange the token for before returning the token (see SI for further details and the video of a test trial provided as Supplementary Material).

### *Condition 1*: Low Token→ Low Food versus High Token = LT (LF vs HT)

The low-value token for the least preferred type of food (dry corn) was placed inside the room and immediately after its return, the subject had to choose between a piece of dry corn and a high-value token. The most economic choice was the token because it gained the parrot a high-value instead of a low-value reward.

### *Condition 2*: Medium Token→ Medium Food vs High Token = MT (MF vs HT)

The medium-value token, associated with a sunflower seed, was offered to the bird. After handing it back, the subject could choose between a sunflower seed and the high-value token. Again, the high token represented the more economic option, however this time the difference in value was less pronounced and therefore this condition was considered to be more challenging than the previous one.

### *Condition 3 (Control)*: Low Token→ Low Food versus Medium Token = LT (LF vs MT)

In this condition, the trial started with a low-value token followed by the choice between a piece of dry corn and a medium-value token. By choosing the token the birds could increase their payoff. At the same time this condition controlled whether the birds were also prepared to exchange their token against another token, if it was not the high-value token (i.e. in case they had a preference for the high token in particular).

### *Condition 4 (Control)*: Medium Token→ Medium Food versus Low Token = MT (MF vs LT)

After handing out the medium-value token, the subject could choose between the sunflower seed and the low-value token. In this case, choosing the token led to a lower payoff than accepting the immediate food.

### *Condition 5 (Control)*: Medium Token→ Medium Food vs Medium Token = MT (MF vs MT)

In this condition, both options i.e. the immediate food (a sunflower seed) and the token offered, had the same value. Choosing the token however led to a delay in payoff and increased the subject’s effort.

### *Condition 6 (Control)*: High Token → High Food versus High Token = HT (HF vs HT)

This condition was identical to *condition 4*, but here the food item and the token were of a high instead of medium value. The most efficient decision was to accept the immediately available piece of walnut.

The conditions *4*–*6* served as controls to test whether the bird applied the rule “always choose the token” or had preferences for the high token in general and chose without assessing the values of the two options.

### Data Analysis

In a binomial distribution, the one-tailed probability of selecting 15 or more out of 20 (or five or fewer out of 20) is p = 0.041, so we considered these upper and lower cut-offs as statistically significant at alpha = 0.05. To test for inter-species differences across test conditions we used the Kruskal-Wallis test since the data were not normally distributed. For post-hoc pairwise comparisons the Dunn-Bonferroni test was used.

### Ethical Standards

All applicable international, national, and/or institutional guidelines for the care and use of animals were followed. In accordance with the German Animal Welfare Act of 25th May 1998, Section V, Article 7 and the Spanish Animal Welfare Act 32/2007 of 7th November 2007, Preliminary Title, Article 3, the study was classified as non-animal experiment and did not require any approval from a relevant body.

## Electronic supplementary material

Supplementary Information

Example trial in Condition 2

## Data Availability

All data generated or analysed during this study are included in this published article (and its Supplementary Information files).
