# Supplementary material for: Economic Decision-Making in Parrots
Source: Sci Rep. 2018 Aug 22;8:12537. doi: 10.1038/s41598-018-30933-5 (PMC6105634; doi:10.1038/s41598-018-30933-5)
Supplement: Supplementary file 1 — Supplementary Information [file 41598_2018_30933_MOESM1_ESM.pdf]

Title: **Economic Decision-Making in Parrots**

SUPPLEMENTARY INFORMATION

Anastasia Krasheninnikova[1,2], Friederike Höner[3], Laurie O'Neill[1,2], Elisabetta Penna[4], Auguste von Bayern[1,2]

[1]Max-Planck-Institute for Ornithology, Eberhard-Gwinner-Str., 82319 Seewiesen, Germany

[2]Max-Planck Comparative Cognition Research Station, Loro Parque Fundacíon, 38400 Puerto de la Cruz, Tenerife, Spain

[3]Biozentrum Grindel and Zoological Museum, Martin-Luther-King-Platz 3, 20146 Hamburg, Germany

[4]Department of Life Science and Systems Biology, University of Turin, Via Accademia Albertina 13, 10123 Turin, Italy

Corresponding author contact: akrashe@orn.mpg.de, avbayern@orn.mpg.de

Procedures

Before starting the present study, the birds were trained to token exchange by handing them a small metal object (a token) through the opening in the window between the two testing compartments and then proffering a cupped hand palm to them. If the bird returned the token through the hole in the Perspex panel covering the opening in the window, and placed it in the experimenter's hand it was rewarded with a small piece of walnut (approx. 0.8g). The token (a metal washer of approx. 3.5 cm in diameter) that was used during this initial training was not used for later training or testing.

Each session consisted of 15-20 trials. The criterion to move onto testing was 15 correct exchanges in 15 trials within one session. Subjects that did not spontaneously exchanged the token after 2 sessions (i.e. 30-40 trials) were shaped. Here, the subject was first rewarded for touching the token, then moving the token, then moving the token in the experimenter's direction until they were able to hand the token back into the experimenter's hand. Animals were trained and tested individually. The food items selected for each bird were three preferred foods for which they were willing to exchange tokens.

Food preference tests

Prior to the start of the token exchange training individual food preferences were determined by providing the birds with a pair of foods and recording which ones were selected over others. Sitting in front of the parrot the experimenter simultaneously presented two different type of food in front of the middle hole in the Perspex panel covering the window opening and then moved them approximately 30 cm apart to the two lateral holes of the panel. The food was considered chosen when the bird put their head through one of the holes to reach the desired food. The unchosen hand was closed and then removed, so only the first chosen item could be obtained.

A preference was considered clear if a parrot selected one food over another potential food in ten out of twelve paired choices conducted in a single session. Using these criteria, we were able to use a piece of walnut (approx. 0.9 g) as most preferred food (high-value reward), a sunflower seed as less preferred food (medium-value reward), and a kernel of dry corn as the least preferred, but still desirable food (low-value reward) for all subjects. To ensure that the least preferred food was still "desirable" it had to be eaten ten times in a row without hesitation in a separate session. Once a food pair was established for each bird, and all subjects learned the token exchange procedure, they then learned to associate a particular food with a particular token. The three tokens used were a 3 cm diameter piece of PVC pipe, a metal bracket 3x3 cm long, and a 3.5cm long metal loop (Fig.S1). The birds were acclimated to these objects since they were used for a previous token training (Krasheninnikova et al., unpublished data) but were randomly assigned with new values for the present study.

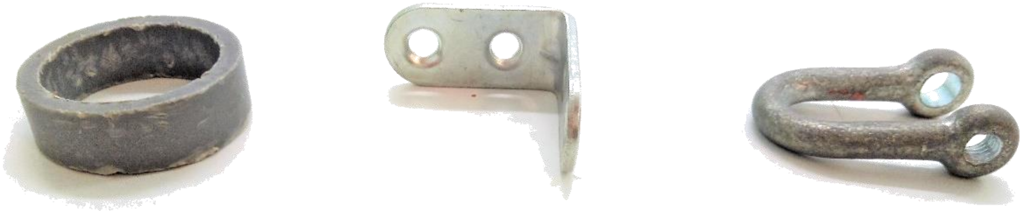

Fig.S1: Three tokens used for the test (from left to the right: a piece of PVC pipe as high-value token, a metal bracket as medium-value token, and a metal loop as low-value token).

Token association training

Following the token association procedure from previous studies, the birds were taught to associate each of the tokens with a particular food by placing three of each token (nine tokens in total) in a random arrangement on the table in animal's testing compartment. As birds gave the tokens to the experimenter, they received the food associated with that token. If animals associated the tokens with the foods, we assumed that they would first hand out the three tokens associated with the high-value food followed by the three tokens associated with the medium-value food and finally, the three associated with the low-value food. We considered association training complete when the subject returned all the tokens in the correct sequence in three consecutive 9-trial training sessions. Two African grey parrots never reached the criterion and were excluded from the test.

General testing procedure

After reaching the training criterion the subjects proceeded to the six test conditions. Two trials of each condition were presented in a pseudo-randomised and counter-balanced manner within the same session (10 sessions in total) to control for order or/and experience effects. Each subject received a maximum of two sessions a day with a time out of at least two hours in between. The subjects were placed into a testing chamber, the experimenter was sitting in front of the subject in another testing chamber, only separated by a window with an opening covered with a Perspex panel with three holes in a row, approximately 8cm apart (Fig.S2). Each test session started with a set of all three tokens (high, medium and low) on the table and the subject was asked to return them one by one in the order high, medium and low, to remind the bird of the token values. If the subject failed to

hand out the token in the correct order, the session would be finished and postponed on the following day. However, none of the subjects ever failed this association trial.

After the bird completed the association trial at the beginning of the session, an initial token was handed to the subject by the experimenter through a slot under the Perspex panel. Once the bird returned the initial token into the experimenter's open palm held under the middle hole, the experimenter removed the hand with the initial token and immediately offered a choice between two hands with palms facing up, one holding the food reward associated with the initial token the other holding another token, associated with the higher, the same or the lower food reward, depending on the condition (Fig.S2). The experimenter first held both items in both hands next to each other in front of the middle hole and then slowly moved both hands apart until both items were held out of the subject's reach approximately 15 cm away from the window opening and approximately 30 cm apart. Thus, a bird needed to unambiguously reach through one of the outer two holes in a Perspex panel for one item. A choice was recorded when a bird put its head through the hole in the Perspex panel in front of an item and reached out for the item. The experimenter then gave the item to the bird and immediately moved away the other hand holding the other item. If the animal selected the token, it could then immediately exchange it, through the middle hole, for the associated food item.

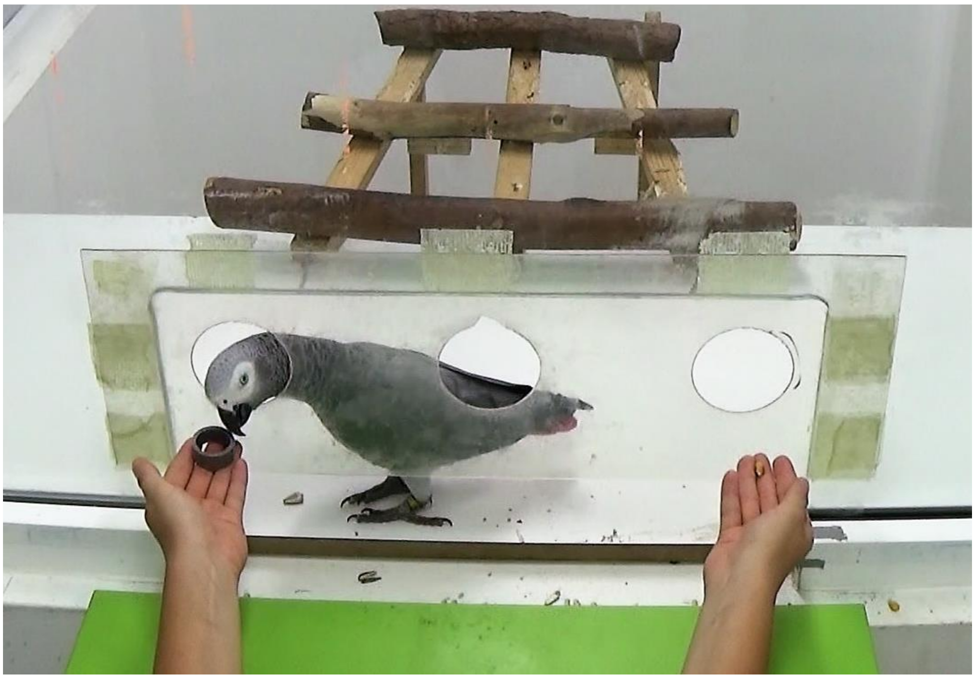

Fig.S2: The subject, an African grey parrot (*Psittacus erithacus*) in the test condition 1, making a choice between a low-value food (dry corn) in the experimenter's right hand and a high-value token (associated with a piece of walnut) in the left hand.

Table S1: Overview on the number of sessions needed to reach the token association training

Species	Individual	# of sessions
Great green macaws	Acorn	11
	Alba	13
	Enya	13
	Hagrid	10
	Hazel	9
	Luna	13
	Madame Maxime	19
	Rita	29
Blue-throated macaws	Baloo	17
	Captain Jack	30
	Charlie	17
	Lady	28
	Long John	9
	Mowgli	30
	Mr Huang	9
	Paco	9
Blue-headed macaws	Andromeda	8
	Callisto	5
	Lupita	31
	Mars	13
	Mercury	10
	Neptune	7
	Saturn	9
	Venus	7
African grey parrots	Bella	17
	Jack	14
	Jelo	12
	Kimmi*	-
	Lizzy	18
	Nikki	26
	Nina *	-
	Sensei	12

*individuals who did not reach the training criterion after 35 sessions and were excluded from the experiment; the subjects highlighted in italics proceeded with the experiment after having reached 30 association sessions without meeting the association training criterion, but only mixing up one middle- and one low-value token after handing out three high-value tokens first and two middle-value tokens first in at least five consecutive sessions.
